# Receiving a hug is associated with the attenuation of negative mood that occurs on days with interpersonal conflict

**DOI:** 10.1371/journal.pone.0203522

**Published:** 2018-10-03

**Authors:** Michael L. M. Murphy, Denise Janicki-Deverts, Sheldon Cohen

**Affiliations:** 1 Department of Psychology, Carnegie Mellon University, Pittsburgh, Pennsylvania, United States of America; 2 School of Dental Medicine, University of Pittsburgh, Pittsburgh, Pennsylvania, United States of America; Arizona State University, UNITED STATES

## Abstract

Interpersonal touch is emerging as an important topic in the study of adult relationships, with recent research showing that such behaviors can promote better relationship functioning and individual well-being. This investigation considers whether being hugged is associated with reduced conflict-related decreases in positive affect and increases in negative affect as well as whether these associations differ between women and men. A sample of 404 adults were interviewed every night for 14 consecutive days about their conflicts, hug receipt, and positive and negative affect. Results indicated that there was an interaction between hug receipt and conflict exposure such that receiving a hug was associated with a smaller conflict-related decrease in positive affect and a smaller conflict-related increase in negative affect when assessed concurrently. Hug receipt was also prospectively associated with a smaller conflict-related increase in next day negative affect but was not associated with next day positive affect. Associations between hug receipt and conflict-related changes in affect did not differ between women and men, between individuals who were married or in a marital-like relationship and those who were not, or as a function of individual differences in baseline perceived social support. While correlational, these results are consistent with the hypothesis that hugs buffer against deleterious changes in affect associated with experiencing interpersonal conflict. Possible mechanisms through which hugs facilitate positive adaptation to conflict are discussed.

## Introduction

Non-sexual interpersonal touch is emerging as an important topic in the study of adult social relationships (for reviews, see [1–3]). Interpersonal touch can be defined as touch behaviors (e.g., hugging and holding hands) that are used to communicate affection or are generally thought to indicate affection [3]. Enthusiasm for this topic is bolstered by multiple lines of converging evidence suggesting that individuals who engage more frequently in interpersonal touch enjoy better physical, psychological, and relational health (e.g., [4–8]). Mechanistically, theorists have proposed that one of the key pathways through which interpersonal touch benefits well-being is by helping buffer against the deleterious consequences of psychological stress [3].

The theory that interpersonal touch improves well-being by acting as a general stress buffer has garnered a fair amount of empirical support. When exposed to a variety of different experimental laboratory stress tasks, individuals assigned to various interpersonal touch manipulations with romantic partners report less distress [9] and show reduced cardiovascular reactivity [10], cortisol secretion [11], and activation of brain regions associated with emotional and behavioral threat [12] compared to those who did not engage in interpersonal touch with their partners. Moreover, while much less studied, the stress-buffering effects of interpersonal touch do not necessarily appear to be limited to touch provided by a romantic partner. For example, in the previously mentioned study examining activation of brain regions associated with threat [12], individuals assigned to a touch condition with a stranger rather than their partner also showed less threat-related neural activation during exposure to a laboratory stressor.

While much of the research to date on touch as a stress buffer has considered stress broadly, there is reason to believe that touch might be a particularly effective buffer of interpersonal conflict more specifically. This possibility holds important potential implications for health and well-being because conflicts with others are associated with a large range of deleterious outcomes, including psychological distress [13], dysregulation of important physiological systems [14], and increased risk for psychiatric illnesses [15], suicide [16], and physical morbidities [17, 18]. Conceptually, touch may buffer against these consequences by promoting a number of positive interpersonal processes thought to communicate care and inclusion and be protective in the face of conflict [3]. In particular, interpersonal touch is associated with increased attachment security, greater perceived partner support, enhanced intimacy, higher relationship satisfaction, and easier conflict resolution [8, 9, 19, 20]. However, the generalizability of this research is limited insofar as studies have largely focused on the role of touch in romantic relationships. Furthermore, much of the research on interpersonal touch has examined behaviors more relevant to romantic relationships than broader social relationships, such as hand-holding, backrubs, kissing, and general self-reports of behavioral intimacy [2]. To address these issues, here we focus on hugs–a relatively common support behavior that individuals engage in with a wide range of social partners–received by any member of a person’s social network.

Another issue in the literature on interpersonal touch is that most studies considering the stress-buffering effects of touch have focused exclusively on women, suggesting at least an implicit assumption that women benefit from touch more than men. The few studies that address possible differences between men and women have not found consistent sex differences in either psychological or physiological responses to touch (e.g., [7, 10, 21]). However, two of these studies [7, 21] had relatively small sample sizes, reducing the power to reliably detect sex differences. One of the studies [10] had a larger sample size, but the nature, duration, and intensity of the manipulation used in this study makes it difficult to disentangle the effects of interpersonal touch itself from intimate verbal interactions and induced romantic feelings [2]. As such, whether the benefits of hugs depend on an individual’s sex remains an open question.

Here, we studied 404 healthy adult men and women who were interviewed every evening for 14 consecutive days about their experiences of social conflict, receipt of hugs, and positive and negative affect. An earlier analysis of these data [4] found that individuals who reported being hugged on more days were protected from the risk for infectious disease associated with reporting more days of conflict. These findings reflect the potential effects of individual differences in conflict occurrence and hug receipt. The present analyses focus on how within person day-to-day changes in the experience of conflict and hug receipt are related to changes in positive and negative affect measured on the same day (concurrently) as well as on the subsequent day (prospectively).

We focused on positive and negative affect because an individual’s emotional response to stress is thought to be a key pathway connecting the experience of stress to health and well-being [22]. Numerous studies have linked interpersonal conflict to increased negative affect (e.g., [13, 23, 24]). Greater levels of negative affect, in turn, are associated with increased risk for psychiatric and physical morbidities [25, 26]. Conflicts are also associated with decreased positive affect (e.g., [23, 24, 27]), a change that is similarly thought to put people at increased risk for poorer health and well-being [28].

We first sought to replicate previous studies of the association between conflict and affect. In line with this aim, we predicted that experiencing interpersonal conflict would be associated with within person increases in negative affect and decreases in positive affect assessed both concurrently on the same day as well as prospectively on the next day. The primary aim of this work was then to examine whether receiving a hug was associated with less conflict-related distress. Regarding this aim, we hypothesized that hug receipt would be associated with smaller conflict-related increases in negative affect and decreases in positive affect (i.e., a conflict × hug interaction). Finally, we also explored whether men and women differed in the extent to which hugs were associated with less conflict-related changes in affect (i.e., a conflict × hug × sex interaction).

## Materials and methods

### Participants

Data for this project were drawn from two archived viral-challenge studies conducted by our group. Both studies followed a similar protocol. These procedures included a physical exam, questionnaire assessments of demographics, and two weeks of evening telephone interviews assessing daily interpersonal interactions, hugging, and affect. Both parent studies followed these procedures and then exposed participants to a common cold virus; the data reported here are observational and derive from variables collected during study baseline (before the viral exposure). Through the support of the NIH, these data and supporting information are available online at the Common Cold Project website (www.commoncoldproject.com). The two studies included in this article are the Pittsburgh Mind-Body Center Study (PMBC; *N* = 193), conducted between 2000 and 2004; and the Pittsburgh Cold Study 3 (PCS3; *N* = 213), conducted between 2007 and 2011. Although the Common Cold Project website includes three daily interview studies, only PMBC and PCS3 contained questions about hugging.

Volunteers for both studies were recruited from the greater Pittsburgh, PA area via newspaper advertisements and community postings. To be eligible to participate, individuals in PMBC had to be between 21 and 55 years old; individuals in PCS3 had to be between 18 and 55 years old. Additionally, all participants had to be in “good general health” as determined through a medical history and physician-conducted physical exam. “Good general health” was defined as having no history of psychiatric illness, major nasal or otological surgery, asthma, or cardiovascular disease; having normal clinical profiles from urinalysis and blood chemistry measures; not being seropositive for HIV; and not taking regular medications other than oral contraceptives. Individuals were also ineligible to participate if they were pregnant or lactating. All participants provided written informed consent and received an honorarium of $800 (PMBC) or $1000 (PCS3) for their participation in the parent studies. The institutional review boards at Carnegie Mellon University and the University of Pittsburgh approved both studies.

In total, 406 individuals completed PMBC and PCS3. Two participants from PMBC were excluded from the analyses reported here due to missing covariate data, providing a final combined sample size of 404. Using the software G*Power [29], we estimated that this sample size provided 98.96% power to detect a standardized effect size of *r* = .21, the typical effect size found across social psychological science [30]. Note that this power analysis is based on a sample of 404 individuals, and does not account for the repeated within person measurements totaling 5,622 person-days of observation. As such, this power estimate is likely an underrepresentation of the actual statistical power in this study. On average, participants were 33.45 years old (SD = 10.50) and had completed 13.92 years of school (SD = 2.05). There were 217 (54%) men and 187 (46%) women. Two hundred and forty-eight individuals (61%) identified as White, while 156 individuals (39%) identified as non-White (130 of the 156 non-White individuals identified as Black). Ninety-eight participants (24%) reported being currently married or in a marital-like relationship. Full descriptive information regarding the sample can be found in Table 1.

**Table 1:** Descriptive statistics for combined pittsburgh mind body center and pittsburgh cold study 3 samples (*N* = 404).

Variables assessed at baseline	*N*	Range	Mean (SD)
Pittsburgh Mind Body Center Participants	191		
Pittsburgh Cold Study 3 Participants	213		
Male Participants	217		
Female Participants	187		
White Participants	248		
Non-White Participants	156		
Currently Married Participants	98		
Not Currently Married Participants	306		
Age (years)		18–55	33.45 (10.50)
Education Attainment (years)		10–20	13.92 (2.05)
Perceived social support		6–36	29.21 (5.57)
**Variables assessed daily**			
Positive Affect		2.71–24.00	14.72 (4.21)
Negative Affect		0.00–16.79	3.00 (2.76)
Social Interactions Per Day		0.07–14.29	3.54 (1.90)
Days with Interpersonal Conflict		0–12	1.93 (2.09)
Days Participants Received a Hug		0–14	8.66 (4.74)

### Procedures

After completing the screening interview and physical exam, individuals who were eligible to participate were enrolled into the study. They then completed baseline questionnaires regarding demographics and perceived social support. One to three weeks after this baseline session, participants were interviewed via telephone every evening at approximately the same time for 14 consecutive days. Each evening interview included questions about social activities and partners, experiences of interpersonal conflict, whether participants had received a hug, and participants’ mood that day. In total, there were 5,622 person-days of observations available to analyze (404 participants × 14 days of observations– 34 missing interview days).

### Measures

#### Social partners, conflicts, and hugs

During each daily telephone interview, participants were asked whether they engaged in each of five different types of activities with other people during the approximately 24 hours since the previous interview. They were also asked to provide initials for the individuals they interacted with to enable tracking unique interpersonal interactions. The five categories were eating (e.g., having a meal, dessert, or cup of coffee), leisure activities at home (e.g., watching TV, reading, playing a game), leisure activities away from home (e.g., going to a movie, going to a sporting event, or going for a walk or hike), work around the house (e.g., yard work, home improvements, cleaning, laundry, paperwork), and family or personal errands (e.g., grocery shopping, going to the doctor, taking the children somewhere). In addition to these five activities, participants were also asked two broader questions about social interactions occurring in other domains not covered in the five specific questions, including work, school, and organizations (the interview scripts are available online at https://www.cmu.edu/common-cold-project/measures-by-study/daily-interviews/index.html). These seven questions were used to calculate the total number of unique people that individuals interacted with each day for use as a control variable. Participants were also asked each evening whether they had experienced any interpersonal tension or conflict since the previous day’s interview (0 = “*no*,” 1 = “*yes*”) and whether anyone had hugged them since the previous day’s interview (0 = “*no*,” 1 = “*yes*”).

#### Daily positive and negative affect

On each of the 14 interview evenings, participants were asked to rate the extent to which various mood adjectives described how they had felt since waking up that morning. The mood assessment included three components of positive affect and three components of negative affect. The mood adjectives were derived from a previously reported factor analysis [31], and have been used in other studies by our group to form composite indices of positive and negative affect (e.g., [32, 33]). Ratings on the individual mood adjectives were made on a 5-point scale ranging from 0 = “*haven’t felt that way at all today*,” to 4 = “*felt that way a lot today*.” Positive mood states assessed included calm (“calm” and “at ease”), well-being (“happy” and “cheerful”), and vigor (“full of pep” and “lively”). Negative mood states assessed included anger (“hostile” and “angry”), anxiety (“on edge” and “tense”), and depression (“sad” and “unhappy”). A daily positive affect composite variable was created by averaging scores for the calm, well-being, and vigor scales. Internal consistencies for the 14 daily positive affect variables were good (Cronbach’s *α*-values ranged from 0.83 to 0.90). The daily negative affect composite variable was created by averaging scores for the anxiety, depression, and anger scales. Internal consistencies for the 14 daily negative affect variables were also good (Cronbach’s *α*-values ranged from 0.85 to 0.90). Positive and negative affect scores were inversely related across the 14 interview days, with within-day correlations ranging from *r* = -0.357, *p* < .001, to *r* = -0.486, *p* < .001.

We also used the software package Mplus [34] to conduct an exploratory factor analysis (EFA) of the 12 mood adjectives accounting for the multilevel within-person clustering in the data. This EFA was run using the weighted least square mean and variance adjusted (WLSMV) estimator, a probit link function, and geomin oblique rotation. We then used the R package nFactors [35, 36] to conduct a parallel analysis [37] with 10,000 generated random correlation matrices to determine the number of factors to retain from the EFA. We retained two factors with sample eigenvalues greater than the 95th percentile of eigenvalues generated in the parallel analysis (see S1 Fig for a plot of the sample eigenvalues along with the 95th percentile eigenvalues obtained from the parallel analysis). Consistent with our approach to form composite indices of positive and negative affect, the six positively valanced mood items all loaded on one “positive affect” factor, and the six negatively valanced mood items all loaded on a second “negative affect” factor. These positive and negative affect factors were negatively correlated, *r* = -0.288, *p* < .001. Bivariate correlations among the 14-day averages of the six assessed mood states can be found in S1 Table. Factor loadings for the two factors are presented in S2 Table.

#### Baseline perceived social support

In order to examine whether individual differences in perceived social support accounted for any observed findings, we assessed support at study baseline (there were no daily measures of perceived support administered, thus it was not possible to examine within person associations with perceived support). Support was measured using the 12-item version of the Interpersonal Support Evaluation List (ISEL [38]). The ISEL-12 (available online at http://www.psy.cmu.edu/~scohen/ISEL12.html) assesses the availability of individuals with whom participants can speak to about problems, can spend time doing things with, and can rely on for material aid if needed. Responses on the individual items were made on a 5-point scale ranging from 0 = “*definitely false*,” to 4 = “*definitely true*.” Negatively stated items indicating low support were first reverse coded, then total perceived social support was calculated by summing the 12 items. Internal consistency for the scale was good (Cronbach’s *α* = 0.82). Baseline perceived support was positively associated with the proportion of interview days individuals reported receiving a hug, *r* = .330, *p* < .001, but not with the proportion of interview days on which individuals reported experiencing conflict, *r* = -0.035, *p* = .484.

#### Control variables

Five between subjects demographic control variables included in all analyses were collected during the baseline screening session. These variables were age (years), sex (male or female), race (coded as White or not-White due to the small number of non-Black racial groups represented), marital status (coded as being currently married or living in a marital-like relationship versus not being currently married or living in a marital-like relationship), and education (number of years of school completed). An additional between subjects covariate indicating whether the participant was in PMBC or PCS3 was also included in all analyses. Furthermore, to rule out the possibility that any observed associations among mood, conflict, and hug receipt were due to conflict and hug receipt merely being indicative of more social contacts, a within subjects covariate indicating the total number of social interactions that individuals reported each day was also included in all analyses. Additionally, individuals’ average number of social contacts and average levels of both positive and negative affect across the 14 interview days were computed and included as between subjects covariates in all models.

### Data analyses

We used the software package HLM 7.03 [39] to evaluate a series of two-level multilevel models examining within person changes in negative or positive affect during the 14-day interview period as a function of receiving a hug, experiencing conflict, and the interaction between receiving a hug and experiencing conflict. We considered the concurrent associations among hugs, conflicts, and negative or positive affect measured on the same day. We also considered prospective lagged associations among hugs and conflicts assessed on the same day and (residual) changes in negative and positive affect from that day to the next. Finally, to address whether affect might precede changes in conflict and hugs, we examined prospective lagged associations between positive and negative affect on the same day and (residual) changes in the likelihood of either receiving a hug or experiencing conflict on the following day.

In all models, within person changes in either same day or next day positive or negative affect were initially predicted at level-1 by whether participants received a hug or experienced conflict (in describing our analyses and reporting our results we use the term “predict” in the conventional statistical sense, not to imply that we are demonstrating a causal mechanism in this observational study). This analysis allowed us to first evaluate the independent associations between hugs and affect and conflicts and affect (i.e., not conditioned on the interaction between hugs and conflicts). Then, to test whether there was an interaction between hugs and conflicts in predicting affect, we next added the level-1 hug × conflict term to the model. To examine the extent to which any associations among hugs, conflicts, and affect were primarily accounted for by hugs received by those who were married or in a marital-like relationship, in subsequent models we also assessed whether marital status interacted with hug receipt and conflict exposure in predicting affect. For this, we added terms for marital status × hug, marital status × conflict, and marital status × hug × conflict to the model testing the hug × conflict interaction. Additionally, to examine the extent to which any associations among hugs, conflicts, and affect were accounted for by individual differences in baseline perceived social support, we conducted separate analyses including model terms for support, support × hug, support × conflict, and support × hug × conflict.

Next, in separate models, we explored whether individual differences in sex interacted with hug receipt and conflict exposure in predicting affect. For this, we first examined the interactions between sex and hug receipt and between sex and conflict exposure. Then, to test whether there was an interaction among sex, hugs, and conflicts in predicting affect, we next added the hug × conflict and sex × hug × conflict terms to the model.

In all models, we included the within subject variable for how many social interactions participants reported having on a given day as a person mean centered level-1 covariate. Additionally, in the models predicting next day hug receipt or conflict exposure from previous day affect, we included within subject variables for previous day hug receipt and conflict exposure. All models also included a random intercept term and random slope terms for all level-1 variables. We also adjusted all models for individual differences in age, sex, race, marital status, education, study, average number of social partners interacted with across the 14 days, average positive affect across the 14 days, and average negative affect across the 14 days at level-2. We grand mean centered all level-2 variables (for discussions regarding the logic of centering binary variables, see [40–42]). The one exception was sex, which was not centered (0 = female, 1 = male). In the prospective lagged models, we additionally included two person mean centered variables indicating individuals’ previous day positive and negative affect scores at level-1 (i.e., regardless of whether next day negative affect or next day positive affect was being predicted, both positive and negative affect on the previous day were simultaneously adjusted for).

All models were estimated using restricted maximum likelihood. Inferences regarding fixed effects were based on robust standard error estimates. Significant interactions were probed using previously described methods [43]. For continuous outcome variables, we present the unstandardized multilevel model regression coefficients (*β*) for the fixed effects. For binary outcome variables, we present the odds ratios (*OR*) for the fixed effects. For significant interactions, we present unstandardized regression coefficients (*b*) for the simple slopes. We report two-tailed *p*-values and 95% confidence intervals (CI_95_) for all analyses. Finally, we obtained multilevel model effect size estimates (*d*) using previously described methods [44].

## Results

### Preliminary information regarding hugs and conflicts

Most individuals reported receiving a hug on at least one interview day (*N* = 377; 93.32%) and experiencing conflict on at least one interview day (*N* = 279; 69.06%). A multilevel logistic regression analysis predicting hugs from conflicts (with no covariates) revealed that hugs were more likely to occur on days when conflict occurred, *OR* = 1.306, *p* = .011, CI_95_ = [1.062, 1.606] (for a contingency table of the number of person-days in which conflicts and hugs did and did not occur see Table 2).

**Table 2:** Contingency table of the number of person-days on which hugs and conflicts occurred.

	Did not experience conflict	Experienced conflict
**Did not receive hug**	1916 (34.08%)	218 (3.88%)
**Received hug**	2931 (52.13%)	557 (9.91%)

Note: There were 404 participants × 14 interview days = 5656 potential person-days of observations. Data regarding conflicts and hugs were missing on 34 person-days, resulting in a final total of 5622 person-days of observation.

### Experiencing conflict, receiving a hug, and same day affect

#### Negative affect

**Fig 1:**
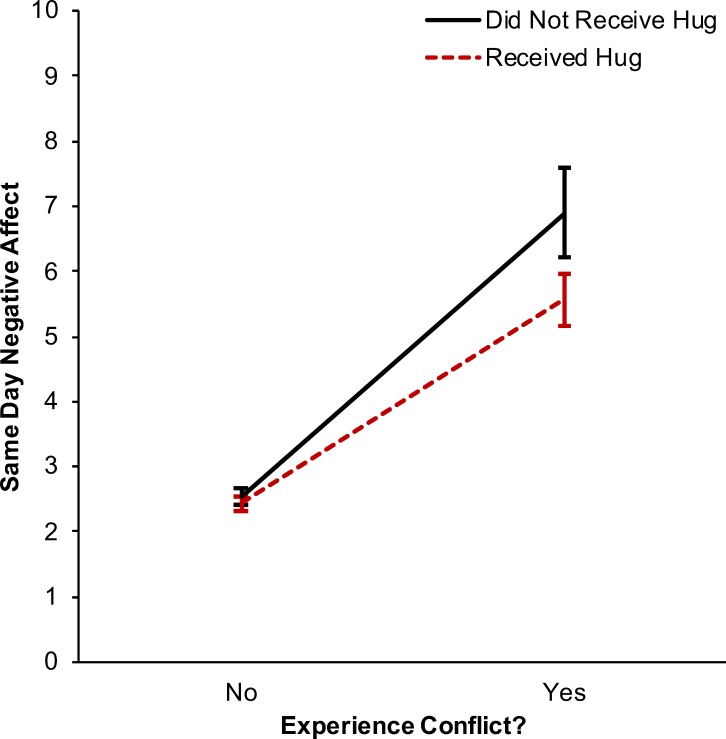
Interaction between hug receipt and conflict in predicting same day negative affect. There was a significant interaction between experiencing conflict and receiving a hug in predicting same day negative affect. When individuals reported conflict without a hug they had higher concurrent negative affect than when they had also received a hug. Error bars represent 95% confidence intervals for the mean predicted same day negative affect values.

On days when individuals reported receiving a hug they had lower negative affect, *β* = -0.240, *p* = .005, CI_95_ = [-0.407, -0.073], *d* = -0.088. Conversely, on days when individuals reported experiencing conflict they had higher negative affect, *β* = 3.455, *p* < .001, CI_95_ = [3.034, 3.877], *d* = 1.271. There was a significant interaction between conflict exposure and hug receipt in predicting same day negative affect, *β* = -1.217, *p* = .002, CI_95_ = [-1.977, -0.457], *d* = -0.453 (Fig 1; see S3 Table for full model results). Simple slopes analyses indicated that on days when individuals reported conflict without a hug they had higher concurrent negative affect than on days when they had also received a hug, *b* = -1.338, *p* < .001, CI_95_ = [-2.080, -0.597], *d* = -0.498. There was no evidence for a three-way interaction among marital status, hug receipt, and conflict exposure in predicting negative affect, *β* = -0.290, *p* = .758, CI_95_ = [-2.135, 1.556], *d* = -0.108. Likewise, there was no evidence for a three-way interaction among individual differences in perceived social support, hug receipt, and conflict exposure in predicting negative affect, *β* = 0.021, *p* = .714, CI_95_ = [-0.091, 0.132], *d* = .008.

#### Positive affect

**Fig 2:**
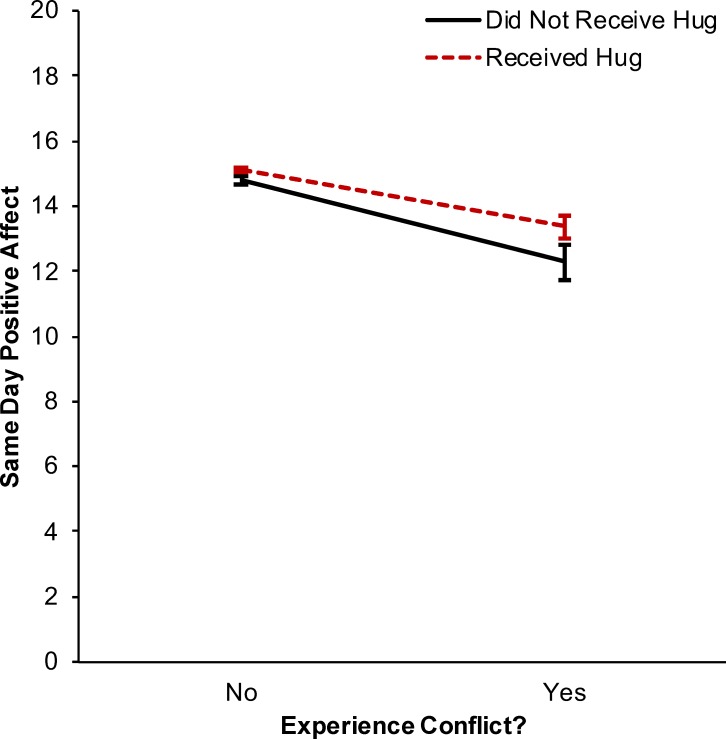
Interaction between hug receipt and conflict in predicting same day positive affect. There was a significant interaction between experiencing conflict and receiving a hug in predicting same day positive affect. When individuals reported conflict without a hug they had lower concurrent positive affect than when they had also received a hug. Error bars represent 95% confidence intervals for the mean predicted same day positive affect values.

On days when individuals reported receiving a hug they had higher positive affect, *β* = 0.406, *p* < .001, CI_95_ = [0.232, 0.581], *d* = 0.136. Conversely, on days when individuals reported experiencing conflict they had lower positive affect, *β* = -1.955, *p* < .001, CI_95_ = [-2.297, -1.614], *d* = -0.654. There was a significant interaction between conflict exposure and hug receipt in predicting same day positive affect, *β* = 0.751, *p* = .020, CI_95_ = [0.121, 1.381], *d* = 0.252 (Fig 2; see S4 Table for full model results). Simple slopes analyses indicated that on days when individuals reported conflict without a hug they had lower concurrent positive affect than on days when they had also received a hug, *b* = 1.085, *p* = .001, CI_95_ = [0.471, 1.700], *d* = 0.365. There was no evidence for a three-way interaction among marital status, hug receipt, and conflict exposure in predicting positive affect, *β* = 0.819, *p* = .244, CI_95_ = [-0.560, 2.197], *d* = 0.275. Likewise, there was no evidence for a three-way interaction among individual differences in perceived social support, hug receipt, and conflict exposure in predicting positive affect, *β* = -0.035, *p* = .517, CI_95_ = [-0.143, 0.072], *d* = -0.012.

### Experiencing conflict, receiving a hug, and next day affect

#### Negative affect

**Fig 3:**
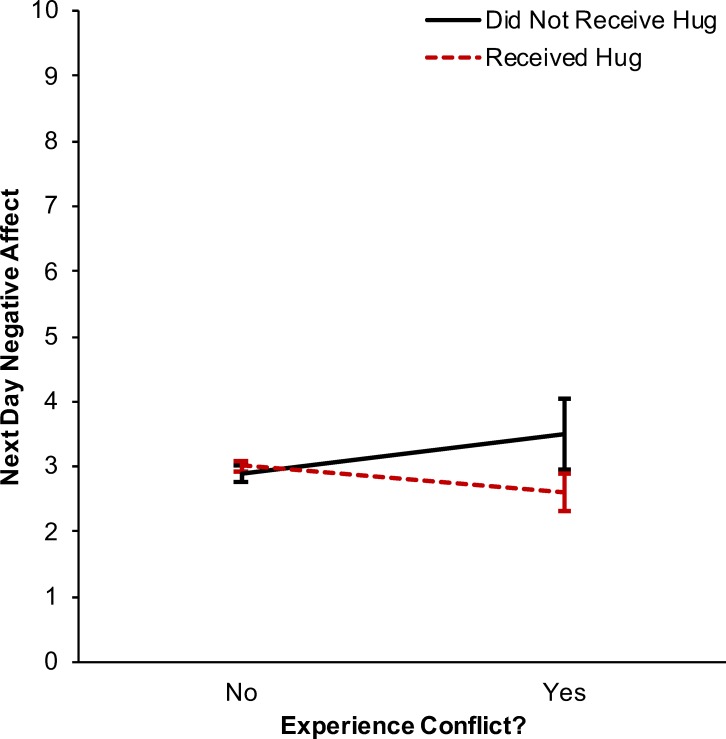
Interaction between hug receipt and conflict in predicting next day negative affect. There was a significant interaction between experiencing conflict and receiving a hug in predicting next day negative affect. When individuals reported conflict without a hug they had higher negative affect on the next day than when they had also received a hug on the previous day. Error bars represent 95% confidence intervals for the mean predicted next day negative affect values.

There was no association between receiving a hug and next day negative affect, *β* = 0.025, *p* = .777, CI_95_ = [-0.149, 0.199], *d* = 0.008. Likewise, there was no association between experiencing conflict and next day negative affect, *β* = -0.152, *p* = .328, CI_95_ = [-0.459, 0.154], *d* = -0.051. There was a significant interaction between conflict exposure and hug receipt in predicting next day negative affect, *β* = -1.022, *p* < .001, CI_95_ = [-1.618, -0.426], *d* = -0.345 (Fig 3; see S5 Table for full model results). Simple slopes analyses indicated that on days when individuals reported conflict without a hug they had higher negative affect the next day than on days when they had also received a hug on the previous day, *b* = -0.896, *p* = .003, CI_95_ = [-1.491, -0.301], *d* = -0.302. There was no evidence for a three-way interaction among marital status, hug receipt, and conflict exposure in predicting negative affect, *β* = 0.344, *p* = .635, CI_95_ = [-1.079, 1.767], *d* = 0.116. Likewise, there was no evidence for a three-way interaction among individual differences in perceived social support, hug receipt, and conflict exposure in predicting negative affect, *β* = -0.050, *p* = .188, CI_95_ = [-0.125, 0.025], *d* = -0.017.

#### Positive affect

There was no association between receiving a hug and next day positive affect, *β* = -0.087, *p* = .297, CI_95_ = [-0.252, 0.077], *d* = -0.029. Likewise, there was no association between experiencing conflict and next day positive affect, *β* = 0.128, *p* = .368, CI_95_ = [-0.151, 0.407], *d* = 0.042. There was also no interaction between conflict exposure and hug receipt in predicting next day positive affect, *β* = 0.401, *p* = .180, CI_95_ = [-0.186, 0.989], *d* = 0.131 (see S6 Table for full model results). Likewise, there was no evidence for a three-way interaction among marital status, hug receipt, and conflict exposure in predicting positive affect, *β* = 0.499, *p* = .463, CI_95_ = [-0.837, 1.835], *d* = 0.163. Finally, there was no evidence for a three-way interaction among individual differences in perceived social support, hug receipt, and conflict exposure in predicting positive affect, *β* = 0.006, *p* = .887, CI_95_ = [-0.073, 0.084], *d* = 0.002.

### Interactions among sex, hugs, conflicts, and affect

Separate multilevel logistic regression analyses predicting hugs and conflicts as a function of sex (with no covariates) revealed that compared to men, women reported both more hugs, *OR* = 2.625, *p* < .001, CI_95_ = [1.740, 3.962], and more conflicts, *OR* = 1.697, *p* < .001, CI_95_ = [1.326, 2.170].

#### Same day affect

There was no interaction between sex and receiving a hug in predicting same day negative affect, *β* = 0.238, *p* = .107, CI_95_ = [-0.320, 0.797], *d* = 0.088. However, there was a significant interaction between sex and receiving a hug in predicting same day positive affect such that women reported a larger increase in positive affect than men on days in which individuals received a hug, *β* = -0.634, *p* < .001, CI_95_ = [-0.931, -0.338], *d* = -0.213. There was tentative evidence suggesting an interaction between sex and experiencing conflict in predicting same day negative affect such that women tended to report a larger increase in negative affect than men on days in which individuals experienced conflict, *β* = -0.702, *p* = .085, CI_95_ = [-1.500, 0.097], *d* = -0.258. There was also a significant interaction between sex and experiencing conflict in predicting same day positive affect such that women reported a larger decrease in positive affect than men on days in which individuals experienced conflict, *β* = 0.740, *p* = .026, CI_95_ = [0.088, 1.392], *d* = 0.248. There was no evidence for a three-way interaction among sex, receiving a hug, and experiencing conflict in predicting same day negative affect, *β* = 0.139, *p* = .856, CI_95_ = [-1.367, 1.644], *d* = 0.052. Likewise, there was no evidence for a three-way interaction among sex, receiving a hug, and experiencing conflict in predicting same day positive affect, *β* = -0.338, *p* = .599, CI_95_ = [-1.601, 0.924], *d* = -0.114.

#### Next day affect

There was no interaction between sex and receiving a hug in predicting next day negative affect, *β* = 0.236, *p* = .126, CI_95_ = [-0.067, 0.539], *d* = 0.079, nor was there an interaction between sex and receiving a hug in predicting next day positive affect, *β* = -0.128, *p* = .386, CI_95_ = [-0.418, 0.162], *d* = -0.042. Likewise, there was no interaction between sex and experiencing conflict in predicting next day negative affect, *β* = 0.242, *p* = .387, CI_95_ = [-0.307, 0.790], *d* = 0.081, nor was there an interaction between sex and experiencing conflict in predicting next day positive affect, *β* = 0.032, *p* = .900, CI_95_ = [-0.463, 0.526], *d* = 0.010. Finally, there was no evidence for a three-way interaction among sex, receiving a hug, and experiencing conflict in predicting next day negative affect, *β* = 0.649, *p* = .277, CI_95_ = [-0.524, 1.822], *d* = 0.219, nor was there evidence for a three-way interaction among sex, receiving a hug, and experiencing conflict in predicting next day positive affect, *β* = -0.828, *p* = .169, CI_95_ = [-2.008, 0.353], *d* = -0.270.

### Prospective associations among previous day affect and next day conflict exposure and hug receipt

As noted above, by adjusting for previous day positive and negative affect in the prospective lagged analyses, we were able to rule out the possibility that previous day affect might account for the observed associations among previous day hug receipt, conflict exposure, and next day affect. However, to test whether previous day affect was additionally associated with the likelihood of receiving a hug or experiencing conflict on the next day, we conducted secondary analyses predicting either hugs or conflicts on the next day as a function of previous day positive and negative affect. Results indicated that conflict exposure was not associated with either previous day positive affect, *OR* = 1.009, *p* = .581, CI_95_ = [0.978, 1.040], or previous day negative affect, *OR* = 1.006, *p* = .726, CI_95_ = [0.974, 1.039]. Likewise, hug receipt was not associated with previous day negative affect, *OR* = 0.999, *p* = .942, CI_95_ = [0.971, 1.028], and was only weakly associated with previous day positive affect, *OR* = 1.028, *p* = .058, CI_95_ = [0.999, 1.055].

## Discussion

We hypothesized that individuals experiencing interpersonal conflicts would have greater negative and lesser positive affect on both the same day and the following day. Moreover, we further predicted that these associations would be attenuated for those receiving hugs on conflict days. Conflicts were independently associated with greater concurrent negative affect and lesser concurrent positive affect, though not with next day negative or positive affect. Receiving a hug on the day of conflict was associated with improved concurrent negative and positive affect and improved next day negative affect compared to days when conflict occurred but no hug was received.

These results were independent of a number of controls included to address a variety of alternative explanations. Controls included in all models were age, sex, race, marital status, education, study, daily numbers of social interactions, 14-day mean numbers of daily social interactions, and 14-day mean levels of both positive and negative affect. All prospective lagged analyses additionally controlled for both previous day positive and negative affect. In additional follow-up analyses, we did not find evidence that associations among hugs, conflicts, and affect varied as a function of marital status. Additionally, in separate analyses, we did not find evidence that associations among hugs, conflicts, and affect varied as a function of individual differences in global perceptions of baseline perceived social support.

Although the primary prospective lagged analyses indicated that the interaction between conflicts and hugs predicted changes in negative affect from one day to the next, secondary prospective analyses did not find an association of previous day affect with the likelihood of receiving a hug or experiencing conflict on the subsequent day. This provides tentative evidence for the hypothesized direction of causation; that is, conflicts and hugs were associated with later changes in affect, but affect was not associated with later changes in conflict and hugs.

We found sex differences in the number of days individuals reported conflicts and hugs. In both cases, women reported more events than men. We also explored whether sex differences exist in the extent to which hugs were associated with less conflict-related change in affect. There were no differences in either the concurrent or the prospective lagged analyses. Thus, our results are consistent with the conclusion that both men and women may benefit equally from being hugged on days when conflict occurs.

While we have interpreted our results as being consistent with the hypothesis that hugs buffer against conflict-related changes in affect, an alternative possibility is that interpersonal conflict interferes with improved affect associated with receiving a hug. The correlational design of our study does not allow us to definitively rule this alternative hypothesis out, however an examination of the results figures along with the magnitudes of the conditional associations obtained from probing the interactions suggests this alternative is less consistent with our data. For the alternative interpretation that conflicts interfere with changes in affect associated with hugging to be plausible, we would expect the effect size estimates for the associations between hug receipt and affect on non-conflict days to be larger than the effect size estimates for the associations between hugs and affect on conflict days. Instead, hugs appear to be associated within person with a larger beneficial change in affect when conflicts occur (*d* = 0.365 for positive affect and *d* = -0.498 for negative affect) compared to when conflicts do not occur (*d* = 0.122 for positive affect and *d* = -0.045 for negative affect). Likewise, conflicts appear more strongly associated with poorer affect on days when hugs do not occur (*d* = -0.843 for positive affect and *d* = 1.618 for negative affect) compared to when hugs do occur (*d* = -0.591 for positive affect and *d* = 1.165 for negative affect). This interpretation is also consistent with numerous studies that have documented stronger stress buffering benefits of social support compared to independent benefits (for reviews, see [45–49]). Nonetheless, experimental studies that manipulate conflict and hug exposure will be needed to test whether hugs causally buffer against deleterious changes in affect due to interpersonal conflict.

Conceptually, the key to understanding why hug receipt was associated with less conflict-related decreases in positive affect and increases in negative affect may be in what hugs convey to recipients. Whereas numerous studies have linked the perception of social support to better outcomes following stressful experiences [46], evidence supporting benefits of actual support provision has been less forthcoming (e.g., [50]). One theory that has gained traction in understanding these disparate findings posits that support provision may be ineffective to the extent that it communicates to receivers that they are not competent to manage stressors [51, 52]. Indeed, support provision that is high in responsiveness–defined as conveying understanding, validation, and care–has been associated with better psychological outcomes [53]. Thus, as discussed in the introduction, interpersonal touch behaviors such as hugs may buffer against stressors such as conflict because they increase perceptions of social support availability by tangibly conveying care and empathy [2, 8, 9, 19] without communicating to receivers that the receivers are ineffective. To test these potential mechanisms in individuals’ natural environments, future studies will need to make use of intensive within-day sampling of interpersonal experiences.

It is unclear why we did not observe any associations among conflicts, hugs, and next day positive affect, especially as previous studies have shown that negative social exchanges impact both negative and positive affect longitudinally (e.g., [24, 27]). One possibility is that mood tends to rebound on days following interpersonal conflict (e.g., [13]), and the associations we report here among conflicts, hugs, and positive affect assessed concurrently were generally weaker than for negative affect. Consequently, we may not have found prospective associations because individuals generally did not show as steep of a decline in positive affect to begin with on conflict days (compared to the magnitude of the observed increases in negative affect), and thus had less of a deficit in positive affect to rebound from on the following day.

Strengths of this study include a large sample size, a large number of repeated assessments of conflict, hugs, and affect, and the inclusion of prospective (lagged) analyses. There were also several limitations. First, although we controlled for a variety of potential confounders, these data are correlational and thus it is still possible that some unidentified factors influenced both predictors and outcomes. That being said, our analyses primarily focused on within person changes which are not confounded by individual difference factors. Another limitation was that participants were not asked about whom they experienced conflict with or whom they received hugs from, nor were they asked about the temporal order in which conflicts and hugs occurred within a given day. This lack of specificity restricted our ability to assess whether hugs were effective buffers because they were given in direct response to conflicts or because they provided a buffer when given prior to conflict. The lack of specificity regarding from whom individuals received hugs also restricted our ability to identify whether hugs from specific types of social partners were more effective than those from others (though we were able to rule out that our findings were attributable primarily to hugs received by individuals who were married or in a marital-like relationship). Future studies using multiple measurements per day (e.g., ecological momentary assessments) will be needed to address these mechanistic issues. Additionally, we did not collect data on conflict severity. It is possible that the interaction among hugs, conflicts, and affect may have been partly attributable to hugs being more likely to occur on days when conflict was less severe. Notwithstanding these limitations, this study contributes to our understanding of the role of interpersonal touch in buffering against deleterious outcomes associated with interpersonal conflict. In particular, our findings from a naturalistic community sample with a large sample size suggest that hugs may be a simple yet effective method of providing support to both men and women experiencing interpersonal distress.

## Supporting information

S1 Fig Scree plot comparing sample eigenvalues with 95th percentile eigenvalues obtained from parallel analysis. A comparison of eigenvalues obtained from the exploratory factor analysis of the daily affect data with the 95th percentile of eigenvalues obtained from a parallel analysis based on 10,000 generated random correlation matrices reveals a two factor solution. Specifically, the six positively valenced affect items each loaded on a “positive affect” factor, and the six negatively valenced affect items each loaded on a “negative affect” factor.
(TIFF)

S1 Table Bivariate correlations among 14-day averages of the six assessed mood states. (DOCX)

S2 Table Factor loadings for two affect factors extracted using EFA. (DOCX)

S3 Table Multilevel model results for predicting concurrent negative affect from hug receipt and conflict exposure not conditioned on the interaction between hugs and conflicts. (DOCX)

S4 Table Multilevel model results for predicting concurrent positive affect from hug receipt and conflict exposure not conditioned on the interaction between hugs and conflicts. (DOCX)

S5 Table Multilevel model results for predicting next day negative affect from hug receipt and conflict exposure not conditioned on the interaction between hugs and conflicts. (DOCX)

S6 Table Multilevel model results for predicting next day positive affect from hug receipt and conflict exposure not conditioned on the interaction between hugs and conflicts. (DOCX)
