# Supplementary figures and images for: Receiving a hug is associated with the attenuation of negative mood that occurs on days with interpersonal conflict
Source: PLoS One. 2018 Oct 3;13(10):e0203522. doi: 10.1371/journal.pone.0203522 (PMC6169869; doi:10.1371/journal.pone.0203522)

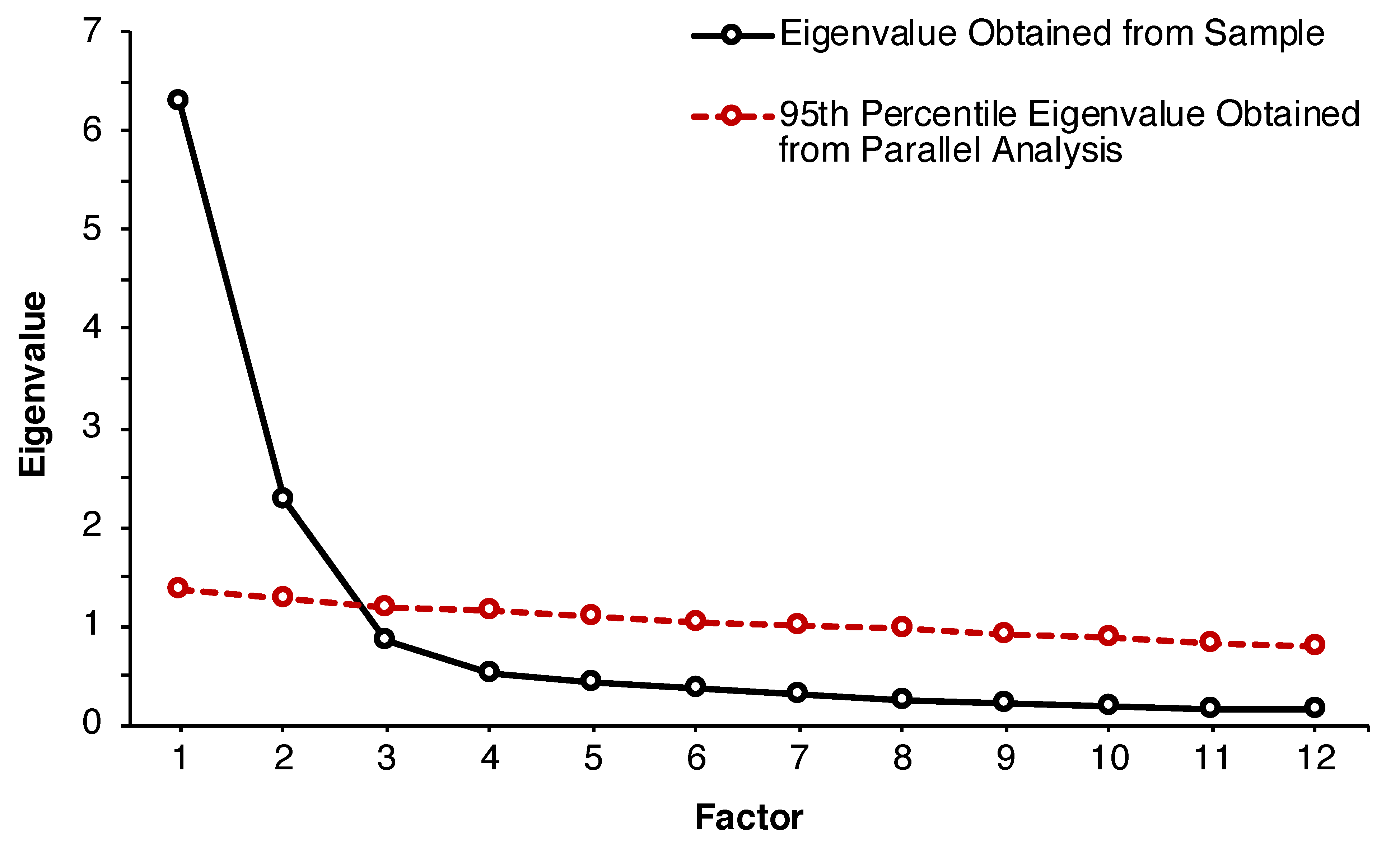

Supplement: S1 Fig — A comparison of eigenvalues obtained from the exploratory factor analysis of the daily affect data with the 95th percentile of eigenvalues obtained from a parallel analysis based on 10,000 generated random correlation matrices reveals a two factor solution. Specifically, the six positively valenced affect items each loaded on a “positive affect” factor, and the six negatively valenced affect items each loaded on a “negative affect” factor. (TIFF) [file pone.0203522.s001.tiff]
