# Supplementary material for: Receiving a hug is associated with the attenuation of negative mood that occurs on days with interpersonal conflict
Source: PLoS One. 2018 Oct 3;13(10):e0203522. doi: 10.1371/journal.pone.0203522 (PMC6169869; doi:10.1371/journal.pone.0203522)
Supplement: S1 Table — (DOCX) [file pone.0203522.s002.docx]

**S1 Table. Bivariate Correlations Among 14-Day Averages of the Six Assessed Mood States**

| **Mood State** | **Calm** | **Well-being** | **Vigor** | **Anger** | **Anxiety** | **Depression** |
| --- | --- | --- | --- | --- | --- | --- |
| Calm | 1 |  |  |  |  |  |
| Well-being | 0.678 | 1 |  |  |  |  |
| Vigor | 0.550 | 0.817 | 1 |  |  |  |
| Anger | -0.368 | -0.240 | -0.156 | 1 |  |  |
| Anxiety | -0.449 | -0.279 | -0.198 | 0.706 | 1 |  |
| Depression | -0.398 | -0.386 | -0.264 | 0.704 | 0.661 | 1 |
