# Supplementary material for: Receiving a hug is associated with the attenuation of negative mood that occurs on days with interpersonal conflict
Source: PLoS One. 2018 Oct 3;13(10):e0203522. doi: 10.1371/journal.pone.0203522 (PMC6169869; doi:10.1371/journal.pone.0203522)
Supplement: S2 Table — (DOCX) [file pone.0203522.s003.docx]

**S2 Table. Factor Loadings for Two Affect Factors Extracted Using EFA**

| **Mood State** | **Positive Affect Factor Loadings** | **Negative Affect Factor Loadings** |
| --- | --- | --- |
| Lively | **0.899** | 0.015 |
| Full of Pep | **0.873** | 0.030 |
| Cheerful | **0.797** | -0.140 |
| Happy | **0.721** | -0.219 |
| At Ease | **0.528** | -0.413 |
| Calm | **0.486** | -0.379 |
| Angry | 0.015 | **0.869** |
| Hostile | 0.100 | **0.858** |
| On Edge | 0.015 | **0.852** |
| Tense | -0.034 | **0.827** |
| Unhappy | -0.158 | **0.813** |
| Sad | -0.158 | **0.765** |

Note: Bolded values indicate which factor the corresponding adjective most strongly loaded on.
