# Supplementary material for: Receiving a hug is associated with the attenuation of negative mood that occurs on days with interpersonal conflict
Source: PLoS One. 2018 Oct 3;13(10):e0203522. doi: 10.1371/journal.pone.0203522 (PMC6169869; doi:10.1371/journal.pone.0203522)
Supplement: S3 Table — (DOCX) [file pone.0203522.s004.docx]

**S3 Table. Multilevel Model Results for Predicting Concurrent Negative Affect from Hug Receipt and Conflict Exposure Not Conditioned on the Interaction Between Hugs and Conflicts**

| **Fixed Effects** | ***β*** | ***p*-value** | **CI_95_** |
| --- | --- | --- | --- |
| Intercept | 2.624 | < .001 | [2.502, 2.746] |
| Sex | 0.053 | .219 | [-0.031, 0.137] |
| Age | -0.003 | .177 | [-0.008, 0.001] |
| Race | 0.061 | .261 | [-0.045, 0.167] |
| Study | -0.001 | .977 | [-0.102, 0.099] |
| Education | 0.016 | .168 | [-0.007, 0.039] |
| Marital Status | -0.039 | .555 | [-0.169, 0.091] |
| Mean Social Interactions | -0.044 | .004 | [-0.073, -0.014] |
| Mean Positive Affect | 0.007 | .169 | [-0.003, 0.017] |
| Mean Negative Affect | 0.900 | < .001 | [0.873, 0.928] |
| Daily Social Interactions | -0.023 | .391 | [-0.075, 0.029] |
| Hug Receipt | -0.240 | .005 | [-0.407, -0.073] |
| Conflict Exposure | 3.455 | < .001 | [3.034, 3.877] |
| Hug × Conflict (tested in subsequent step)^a^ | -1.217 | .002 | [-1.977, -0.457] |
| **Random Effects** | **Variance** | ***χ*^2^(*df*)** | ***p*-value** |
| Intercept | 0.011 | 232.463 (188) | .015 |
| Daily Social Interactions | 0.067 | 333.917 (197) | < .001 |
| Hug Receipt | 0.225 | 331.544 (197) | < .001 |
| Conflict Exposure | 8.982 | 609.301 (197) | < .001 |
| Residual Error | 7.394 |  |  |
| Hug × Conflict (tested in subsequent step)^a^ | 10.169 | 119.351 (58) | < .001 |

^a^The Hug × Conflict interaction term was added to the model after first testing the unconditional associations among hug receipt, conflict exposure, and affect. Except for the Hug × Conflict interaction term, estimates of model parameters presented in this table are based on the model not conditioned by the interaction.
