# Supplementary material for: Receiving a hug is associated with the attenuation of negative mood that occurs on days with interpersonal conflict
Source: PLoS One. 2018 Oct 3;13(10):e0203522. doi: 10.1371/journal.pone.0203522 (PMC6169869; doi:10.1371/journal.pone.0203522)
Supplement: S4 Table — (DOCX) [file pone.0203522.s005.docx]

**S4 Table. Multilevel Model Results for Predicting Concurrent Positive Affect from Hug Receipt and Conflict Exposure Not Conditioned on the Interaction Between Hugs and Conflicts**

| **Fixed Effects** | ***β*** | ***p*-value** | **CI_95_** |
| --- | --- | --- | --- |
| Intercept | 14.749 | < .001 | [14.627, 14.870] |
| Sex | -0.042 | .114 | [-0.094, 0.010] |
| Age | 0.004 | .020 | [0.001, 0.007] |
| Race | -0.012 | .682 | [-0.070, 0.046] |
| Study | -0.004 | .889 | [-0.061, 0.053] |
| Education | -0.003 | .647 | [-0.016, 0.010] |
| Marital Status | -0.039 | .318 | [0.115, 0.038] |
| Mean Social Interactions | 0.006 | .575 | [-0.014, 0.025] |
| Mean Positive Affect | 0.992 | < .001 | [0.986, 0.998] |
| Mean Negative Affect | 0.046 | < .001 | [0.031, 0.060] |
| Daily Social Interactions | 0.216 | < .001 | [0.154, 0.279] |
| Hug Receipt | 0.406 | < .001 | [0.232, 0.581] |
| Conflict Exposure | -1.955 | < .001 | [-2.297, -1.614] |
| Hug × Conflict (tested in subsequent step)^a^ | 0.751 | .020 | [0.121, 1.381] |
| **Random Effects** | **Variance** | ***χ*^2^(*df*)** | ***p*-value** |
| Intercept | 0.006 | 204.816 | .190 |
| Daily Social Interactions | 0.138 | 387.844 | < .001 |
| Hug Receipt | 0.089 | 319.383 | < .001 |
| Conflict Exposure | 3.613 | 348.734 | < .001 |
| Residual Error | 8.926 |  |  |
| Hug × Conflict (tested in subsequent step)^a^ | 3.867 | 57.785 (58) | .483 |

^a^The Hug × Conflict interaction term was added to the model after first testing the unconditional associations among hug receipt, conflict exposure, and affect. Except for the Hug × Conflict interaction term, estimates of model parameters presented in this table are based on the model not conditioned by the interaction.
