# Supplementary material for: Receiving a hug is associated with the attenuation of negative mood that occurs on days with interpersonal conflict
Source: PLoS One. 2018 Oct 3;13(10):e0203522. doi: 10.1371/journal.pone.0203522 (PMC6169869; doi:10.1371/journal.pone.0203522)
Supplement: S5 Table — (DOCX) [file pone.0203522.s006.docx]

**S5 Table. Multilevel Model Results for Predicting Next Day Negative Affect from Hug Receipt and Conflict Exposure Not Conditioned on the Interaction Between Hugs and Conflicts**

| **Fixed Effects** | ***β*** | ***p*-value** | **CI_95_** |
| --- | --- | --- | --- |
| Intercept | 2.950 | < .001 | [2.822, 3.078] |
| Sex | 0.010 | .714 | [-0.045, 0.066] |
| Age | 0.002 | .266 | [-0.001, 0.005] |
| Race | -0.041 | .215 | [-0.106, 0.024] |
| Study | -0.055 | .060 | [-0.112, 0.002] |
| Education | 0.009 | .234 | [-0.006, 0.024] |
| Marital Status | 0.019 | .675 | [-0.071, 0.109] |
| Mean Social Interactions | -0.003 | .741 | [-0.024, 0.017] |
| Mean Positive Affect | -0.001 | .843 | [-0.008, 0.007] |
| Mean Negative Affect | 1.004 | < .001 | [0.988, 1.020] |
| Previous Day Positive Affect | 0.009 | .600 | [-0.024, 0.042] |
| Previous Day Negative Affect | 0.127 | < .001 | [0.085, 0.169] |
| Daily Social Interactions | -0.022 | .435 | [-0.076, 0.033] |
| Previous Day Hug Receipt | 0.025 | .777 | [-0.149, 0.199] |
| Previous Day Conflict Exposure | -0.152 | .328 | [-0.459, 0.154] |
| Hug × Conflict (tested in subsequent step)^a^ | -1.022 | < .001 | [-1.618, -0.426] |
| **Random Effects** | **Variance** | ***χ*^2^(*df*)** | ***p*-value** |
| Intercept | 0.010 | 197.257 (176) | .130 |
| Previous Day Positive Affect | 0.013 | 267.065 (185) | < .001 |
| Previous Day Negative Affect | 0.046 | 300.398 (185) | < .001 |
| Daily Social Interactions | 0.040 | 291.920 (185) | < .001 |
| Hug Receipt | 0.021 | 276.340 (185) | < .001 |
| Conflict Exposure | 0.946 | 249.625 (185) | .001 |
| Residual Error | 8.852 |  |  |
| Hug × Conflict (tested in subsequent step)^a^ | 3.429 | 80.438 (52) | .007 |

^a^The Hug × Conflict interaction term was added to the model after first testing the unconditional associations among hug receipt, conflict exposure, and affect. Except for the Hug × Conflict interaction term, estimates of model parameters presented in this table are based on the model not conditioned by the interaction.
