# Supplementary material for: Receiving a hug is associated with the attenuation of negative mood that occurs on days with interpersonal conflict
Source: PLoS One. 2018 Oct 3;13(10):e0203522. doi: 10.1371/journal.pone.0203522 (PMC6169869; doi:10.1371/journal.pone.0203522)
Supplement: S6 Table — (DOCX) [file pone.0203522.s007.docx]

**S6 Table. Multilevel Model Results for Predicting Next Day Positive Affect from Hug Receipt and Conflict Exposure Not Conditioned on the Interaction Between Hugs and Conflicts**

| **Fixed Effects** | ***β*** | ***p*-value** | **CI_95_** |
| --- | --- | --- | --- |
| Intercept | 14.714 | < .001 | [14.585, 14.843] |
| Sex | -0.021 | .461 | [-0.076, 0.034] |
| Age | 0.001 | .455 | [-0.002, 0.004] |
| Race | 0.027 | .327 | [-0.027, 0.082] |
| Study | 0.051 | .053 | [-0.001, 0.103] |
| Education | 0.006 | .388 | [-0.007, 0.018] |
| Marital Status | -0.008 | .828 | [-0.077, 0.062] |
| Mean Social Interactions | 0.016 | .074 | [-0.002, 0.034] |
| Mean Positive Affect | 1.015 | < .001 | [1.009, 1.022] |
| Mean Negative Affect | 0.004 | .526 | [-0.009, 0.017] |
| Previous Day Positive Affect | 0.079 | < .001 | [0.045, 0.113] |
| Previous Day Negative Affect | -0.023 | .265 | [-0.064, 0.018] |
| Daily Social Interactions | -0.005 | .875 | [-0.062, 0.053] |
| Previous Day Hug Receipt | -0.087 | .297 | [-0.252, 0.077] |
| Previous Day Conflict Exposure | 0.128 | .368 | [-0.151, 0.407] |
| Hug × Conflict (tested subsequently)^a^ | 0.401 | .180 | [-0.186, 0.989] |
| **Random Effects** | **Variance** | ***χ*^2^(*df*)** | ***p*-value** |
| Intercept | 0.013 | 221.603 (176) | .011 |
| Previous Day Positive Affect | 0.014 | 205.914 (185) | .140 |
| Previous Day Negative Affect | 0.036 | 270.664 (185) | < .001 |
| Daily Social Interactions | 0.053 | 306.581 (185) | < .001 |
| Hug Receipt | 0.028 | 266.372 (185) | < .001 |
| Conflict Exposure | 0.161 | 297.400 (185) | < .001 |
| Residual Error | 9.411 | 221.603 (185) | .011 |
| Hug × Conflict (tested subsequently)^a^ | 2.209 | 83.407 (52) | .004 |

^a^The Hug × Conflict interaction term was added to the model after first testing the unconditional associations among hug receipt, conflict exposure, and affect. Except for the Hug × Conflict interaction term, estimates of model parameters presented in this table are based on the model not conditioned by the interaction.
